# Editorial: Endocrine Disruptors in Aquatic Vertebrates

**DOI:** 10.3389/fendo.2021.749030

**Published:** 2021-08-26

**Authors:** Renata Guimarães Moreira, Oliana Carnevali, Fabiana Laura Lo Nostro

**Affiliations:** ^1^Departamento de Fisiologia, Instituto de Biociências, Universidade de São Paulo, São Paulo, Brazil; ^2^Dipartimento Scienze della Vita e dell’Ambiente, Università Politecnica delle Marche, Ancona, Italy; ^3^Laboratorio de Ecotoxicología Acuática, Instituto de Biodiversidad y Biología Experimental y Aplicada (IBBEA), Consejo Nacional de Investigaciones Científicas y Técnicas-Universidad de Buenos Aires, Buenos Aires, Argentina; ^4^Departamento de Biodiversidad y Biología Experimental (DBBE), Facultad de Ciencias Exactas y Naturales (FCEyN), Universidad de Buenos Aires (UBA), Buenos Aires, Argentina

**Keywords:** Aquatic environment, contaminants of emerging concern, biomarkers, xenobiotics effluents, plasticizers, pesticides

As human activities progress, large amounts of substances are produced and released into the aquatic environment. Many of these substances can act as endocrine disrupting chemicals (EDCs) which can interact with the neuroendocrine system of exposed animals, altering their normal physiological function and their progeny.

EDCs are substances capable -at different biological levels- of interfering with hormone synthesis and metabolism, receptors activation, gland structure, behavioral responses and epigenetic changes in exposed organisms. The objective of this Research Topic was i) to better understand the effects of these contaminants in real-world systems, at large spatial scales; ii) to demonstrate the mechanism of action of these EDCs in different phylogenetic groups.

The Research Topic contains 7 contributions, including 5 original research articles and 2 reviews. The original papers investigated the effects of plasticizers, pesticides and effluents using mainly fish as a model.

The review by Celino-Brady et al. showed different approaches and experimental methods used to characterize the effects of emerging EDCs and potential carriers of EDCs, on fish growth and reproduction. *In vivo*, *in vitro* and *in silico* approaches were presented to describe the effects of EDCs. The conclusion of this paper underlines the importance of *in vivo* experiments providing more direct inferences to changes in biological activity, *in vitro* assays allowing the screening of the direct effects of chemicals on a specific tissue or cell, while *in silico* studies are important for robust and rapid screening, optimizing the use and care of animals in research.

The scientific community is aware of the importance of identifying new methods for detecting the presence of EDCs in the environment. The mission of many researchers working in this area is in fact to optimize systems/methods, both *in vitro* and *in vivo*, to quickly identify the presence of EDCs in a reliable, sensitive, and rapid way. This Research Topic welcomed contributions that propose new strategies for the detection of environmental EDCs. In Creusot et al., the human nuclear receptor pregnane X receptor (PXR), a ligand-dependent transcription factor regulating genes involved in xenobiotic metabolism in mammals, was compared with the zebrafish PXR through an *in vitro* approach with reporter cell lines expressing either hPXR or zfPXR. In this context, similarities/differences in response to EDCs, focused on pesticides and numerous steroids, were studied in the human and zebrafish PXR. Different sensitivity of the two receptors depending on the nature of the contaminant was observed. The zfPXR was more sensitive to steroids while hPXR to pesticides. The differences in the modulation of zfPXR in comparison to hPXR suggested zfPXR assays as better candidate to evaluate the potential risks of chemicals in aquatic species.

Along the same lines, it is important to remark the use of transgenic fish. Cooper et al. clearly illustrate the importance of fish developmental stage as a strong tool for estrogen exposure effects and demonstrate the utility of the estrogen sensitive element transgenic zebrafish (ERE-GFP-Casper) for an integrative health analysis of exposure to estrogenic chemical mixtures. This transgenic strain was used to investigate the effects of chronic exposure to WwTW effluent on health and the sensitivity to estrogens at different life stages, using the synthetic estrogen 17a-ethinylestradiol as a positive control. The long-term effects of this chronic exposure included altered gonadal development and sex ratios in favour of females. This method, in addition to being sensitive, has the advantage of identifying the global estrogenic effect of mixtures present in aquatic environments, making it also an effective and inexpensive method.

The endocrine disruptor effects on fish produced by the impact of wastewater in Chilean river basins were analyzed by Barra et al. Although watersheds possess a complexity of multiple stressors, this review showed an integrated approach during the last 15 years analyzing the impacts of pulp and paper mill effluents. In order to explore the reproductive impacts on both introduced and native fish in Chile, the integration of watershed, field, and laboratory studies, together with assessment at different levels of biological organization, allowed to analyze the sustainability of wild fish populations exposed to EDCs. Interestingly, there were clear differences between industrial and urban wastewaters, in particular for the non-native fish. Authors reinforced the need for studies on basic biology of native fish species as natural habitat variability would play a role in modulating the fitness and reproductive outcome, introducing a major risk factor for species conservation and the threat of EDCs.

As an example of complexity of endocrine responses to environmental changes and mitigation, we can highlight the contribution of Ussery et al. They share 30 years of detailed studies on the white sucker (*Catostomus commersonii*) in the Jackfish Bay (north shore of Lake Superior, Canada), where the environmental impact first received effluents from a large bleached-kraft pulp mill, then the installation of secondary waste treatment, changes in the pulp bleaching process and a series of facility closures associated with changing ownership. Although population modeling of the initial reproductive alterations predicted a 30% reduction in population size, the improvements over the last couple of decades help sustain the fish population. This study gives insight that integration studies on fish health, endocrine disruption, chemical exposure, ecosystem recovery and whole organism endpoints provide an important context for the complexity of endocrine responses, species differences, and challenges with extrapolation in an impacted site.

Finally, plasticizers were investigated in two contributions Frenzilli et al. showed the endocrine and genotoxic effects of bisphenol A (BPA) and bisphenol S (BPS) in juvenile brown trout (*Salmo trutta*) using an *in vivo* approach. While BPS affected thyroid function and glucose balance in the exposed fish, BPA showed stronger estrogenic and mutagenic capacity. The results show that both compounds have adverse effects in fish, but with two different mode of action. Negative effects of plasticizers were also detected in fish reproduction. Using zebrafish as a model, the contribution of Godoi et al. showed that Di-isononyl phthalate (DiNP) can trigger follicular atresia in ovaries, affecting the reproductive performance in a nonmonotonic manner. The authors suggest that apoptotic biomarkers may be used as a relevant tool to evaluate the impacts of EDCs on folliculogenesis of aquatic species.

## Author Contributions

All authors listed have made a substantial, direct, and intellectual contribution to the work and approved it for publication.

## Conflict of Interest

The authors declare that the research was conducted in the absence of any commercial or financial relationships that could be construed as a potential conflict of interest.

## Publisher’s Note

All claims expressed in this article are solely those of the authors and do not necessarily represent those of their affiliated organizations, or those of the publisher, the editors and the reviewers. Any product that may be evaluated in this article, or claim that may be made by its manufacturer, is not guaranteed or endorsed by the publisher.

